# Current and Future Directions Using Virtual Avenues for Care Delivery Across the Cancer Continuum

**DOI:** 10.3390/curroncol32050249

**Published:** 2025-04-24

**Authors:** Charlotte T. Lee, Franco Ng, Elizabeth Borycki

**Affiliations:** 1 Daphne Cockwell School of Nursing, Toronto Metropolitan University, Toronto, ON M5B 2K3, Canada; franco.ng@torontomu.ca; 2 School of Health Information Science, University of Victoria, Victoria, BC V8W 2Y2, Canada; emb@uvic.ca

**Keywords:** cancer care delivery, digital health, nursing informatics, nurse-led intervention, oncology nursing, telemedicine, telenursing, virtual oncology care, virtual care

## Abstract

Oncology nurses have long been at the forefront of virtual care, transitioning from telenursing to technology-driven delivery methods that address the evolving needs of cancer patients. Initially developed to overcome barriers to care for rural and underserved populations, virtual care has grown into a critical component of oncology practice. Oncology nurses play a central role in providing timely, personalized, and holistic care, leveraging tools such as remote monitoring, patient-reported outcomes, and mHealth platforms. However, the rapid adoption of virtual care demands a broader focus to sustain its impact. This commentary explores the need to clearly define the role of oncology nurses in virtual care, emphasizing leadership in digital health, the integration of hybrid care models, and workforce training. By addressing these priorities, virtual care can continue to enhance patient outcomes, strengthen nursing-led interventions, and expand the scope of oncology nursing, positioning it as an essential and enduring facet of cancer care delivery.

## 1. Introduction

Oncology nurses have long been at the forefront of providing virtual care, evolving from telephone-based telenursing to sophisticated digital health interventions. Initially designed to improve access for rural and underserved populations, virtual care has now become a fundamental part of cancer care delivery [1]. Virtual care can be defined as or refers to “any interaction between patients and/or members of their circle of care, occurring remotely, using any forms of communication or information technologies, with the aim of facilitating or maximizing the quality and effectiveness of patient care [2] (p. 4)”. Over time, oncology nurses have leveraged virtual platforms to manage symptoms, provide psychosocial support, and integrate patient-reported outcomes (PROs) into personalized care plans [3].

The rapid expansion of virtual oncology care was accelerated by the COVID-19 pandemic, with outpatient care pivoting to virtual formats within days of the World Health Organization’s declaration of the pandemic in 2020. In Ontario, for example, 67% of outpatient oncology encounters shifted to virtual platforms between March and May in 2020, while in Alberta, 37.3% of outpatient visits were virtual between April and June 2020 [4,5]. Though initially a response to a crisis, virtual care has since solidified its place in routine oncology practice and is a preferred mode of care delivery for many patients and providers [5]. For patients, virtual care has improved patient access to care, accessibility of care (for the disabled and those living in geographically distant or isolated areas), decreased wait times, and reduced costs associated with travel [6].

Despite its advantages, virtual oncology nursing continues to evolve as an area of practice, with opportunities to enhance its integration into cancer care. Increasingly, hybrid models that combine virtual and in-person care are emerging as a preferred approach, allowing oncology nurses to optimize symptom management, streamline follow-up visits, and maintain strong patient–provider relationships. While nurses have played a central role in virtual care delivery, their contributions are not always fully recognized within digital health strategies [7], nor are they fully included as stakeholders in the design, implementation, and maintenance of such technological systems of care.

To better understand the current and future roles of oncology nurses as stakeholders in virtual care, this commentary explores the evidence surrounding oncology virtual care and its impacts, and identifies key areas for improvement, particularly regarding the virtual oncology nurses’ role clarity, training gaps, and model-of-care demands. More importantly, this commentary identifies how virtual oncology nursing can be further optimized to enhance patient-centered cancer care while advancing the profession.

## 2. Integration of Virtual Care in Oncology Nursing

The evolution of virtual oncology nursing has closely followed broader advancements in telehealth and virtual nursing. While the dedicated literature on the historical development of oncology-specific virtual nursing is limited, oncology nurses have adapted and integrated virtual modalities in parallel with the broader healthcare industry’s shift toward digital health. Much of what is known about virtual oncology nursing is embedded within the evolution of technologies that are part of telehealth, virtual chronic disease management, and remote symptom monitoring.

Early forms of virtual nursing were grounded in telenursing, where nurses provided remote support via telephone triage, symptom management, and patient education [8]. One of the first documented instances of telenursing occurred in 1974 when Mary Quinn, RN, provided remote nursing care at Logan Airport through Boston Hospital’s telemedicine center, marking the emergence of virtual nursing as a distinct practice [9].

Over time, the use of virtual nursing practice spread throughout the healthcare system, leading to the need for practice guidelines. In 2000, the College of Registered Nurses of Nova Scotia published its first Practice Guidelines for Telenursing, outlining the scope of practice, liability, confidentiality, and standards of care for nurses providing virtual care. The document was updated in 2014 to reflect advances in digital health, though its clinical guidance remained focused primarily on communication skills rather than direct patient care approaches [9]. These guidelines marked an early attempt to define professional expectations for nurses working in virtual settings and highlighted the need for ongoing competency development as telehealth expanded.

While telemedicine initially centered around physician-led care, oncology nurses began using telephone and videoconferencing to provide symptom triage and supportive care in rural and remote settings. Over time, advancements such as electronic patient portals, electronic health records (EHRs), and mobile health (mHealth) applications further expanded nurses’ ability to deliver virtual care. These technologies allowed oncology nurses to track patient-reported outcomes (PROs), monitor chemotherapy side effects, and provide psychosocial support without requiring in-person visits [3]. However, it was found that virtual care alone could not fully replace some hands-on nursing assessments, and this led to hybrid models being developed that blend virtual approaches with periodic in-person visits to optimize oncology nursing care [10,11,12,13].

The COVID-19 pandemic acted as a major catalyst for the rapid expansion of virtual care across all healthcare settings (including oncology nursing). In 2020, 27% of nurses in Canada consulted with patients via videoconferencing, compared to just 3% in 2017 [7]. In specialty clinics, 81% of nurses engaged in virtual consultations, and 66% provided e-visits through secure email platforms [1]. The shift demonstrated how rapidly virtual care could be integrated into oncology nursing, leading to lasting changes in cancer care delivery [5].

While the benefits of virtual oncology nursing, including greater accessibility, cost savings, and improved symptom management, became well documented, the full integration of virtual oncology nursing approaches into existing practice continues to remain understudied [14,15]. Virtual care in Canada has expanded rapidly, particularly during the COVID-19 pandemic, transforming healthcare delivery across various settings [1]. While multiple models of virtual care exist, including those led by nurses, much of the initial expansion was physician-driven, with physicians accounting for the majority of virtual visits in the early stages of implementation [1]. Between April 2020 and March 2021, the number of virtual services provided by physicians increased nearly fourfold, and up to 90% of physicians adopted virtual care across various provinces [1]. Despite this rapid uptake, nurses have also been central to virtual care delivery, particularly in chronic disease management, symptom monitoring, and palliative care. The Canadian Nurses Association (CNA) has recognized the challenges of virtual care adoption, highlighting that while its use has increased, many nurses do not feel adequately trained to use digital health technologies effectively [7]. Even as entry-to-practice nursing informatics competencies have been developed, published, and implemented as part of undergraduate nursing education recommendations [16,17], many nurses still report feeling underprepared to use virtual care tools effectively. Several factors contribute to this lack of preparedness. Nurses often cite insufficient training and limited exposure to telehealth tools during both pre-licensure education and clinical orientation programs [7,10,18]. The rapid rollout of virtual platforms during the COVID-19 pandemic further compounded these gaps, leaving many nurses to adapt on the job without structured support [4]. Usability challenges, such as navigating complex user interfaces, switching between multiple systems, and concerns over data security, also impact confidence and competence [19,20,21]. Additionally, nurses have cited increased workloads and unclear digital care protocols as practical barriers to integrating virtual technologies into routine practice [15]. These findings suggest that enhancing nurses’ readiness for virtual care requires not only digital literacy training but also supportive system design, time allocation, and organizational investment in workflow integration.

## 3. Broader Context of Virtual Nursing Practice

Although this commentary focuses on oncology, virtual nursing has been shown to be applicable across a wide range of care settings. For instance, in primary care, nurses deliver chronic disease management, sexual health services, and preventive screening using virtual means [22]. In hospital avoidance strategies, nurses use telehealth technologies for remote triage, patient monitoring, and early discharge follow-up, thereby reducing emergency department visits and unplanned admissions [23]. Additionally, virtual nursing is being explored in outpatient rehabilitation, mental health, and aged care settings, where it supports continuity of care and patient self-management [24]. This wide-reaching applicability illustrates the growing integration of virtual nursing across various levels of care, including preventive, acute, and chronic care, and across community, outpatient, and institutional settings. Nurses are not only users of virtual care technologies, but increasingly act as leaders, educators, and decision-makers in digital health initiatives. They support technology adoption, ensure equitable access, and shape digital workflows to enhance care delivery.

A 2020 survey by the CNA, the Canadian Nursing Informatics Association, and Canada Health Infoway revealed that only 60% of nurses using virtual care technologies felt they had the necessary knowledge and skills [7]. This underscores the need for structured digital health education and professional development tailored to oncology nursing, to ensure oncology nurses are equipped with the competencies to integrate virtual care seamlessly into their practice.

## 4. Current Evidence of Oncology Nurses Providing Virtual Care

Virtual oncology nursing has expanded across the cancer care continuum, integrating telehealth, digital health platforms, and remote symptom monitoring to enhance accessibility and patient-centered care in several key areas. This current evidence showcases and demonstrates how oncology nurses contribute to symptom management, psychosocial support, palliative care, and large-scale virtual care models.

### 4.1. Symptom Management

Oncology nurses play a critical role in virtual symptom management, leveraging digital tools and remote monitoring platforms to detect complications early and optimize patient care. The Pan-Canadian Oncology Symptom Triage and Remote Support (COSTaRS) guides have standardized telephone-based symptom management, equipping oncology nurses with evidence-based tools to provide consistent, high-quality care remotely [25]. These guides, developed using rigorous systematic reviews and validated by oncology nurses nationwide, support consistent and high-quality symptom management for patients undergoing cancer treatment.

The integration of patient-reported outcomes (PROs) and remote patient monitoring (RPM) has further enhanced oncology nursing practice. A systematic review by Wang et al. (2023) demonstrated that PRO-tracking software improved symptom control, emotional well-being, and self-efficacy in cancer patients [3]. The study highlighted that nurses play a central role in integrating RPMs into clinical oncology care, particularly in monitoring symptoms and triggering timely interventions. Nurse-led RPMs models, such as those incorporating automated alerts to clinical teams, were associated with greater improvements in patient outcomes. Similarly, a real-world study across 33 cancer centers in France and Belgium reinforced these findings, showing that 94.6% of symptom alerts led to improvement within two weeks, with 80% of alerts managed directly by oncology nurses [26].

Technological advancements such as mobile health (mHealth) applications have also facilitated proactive symptom management. Shi et al. (2024) examined nurse-led mobile health interventions for self-monitoring, finding that digital engagement reduced unplanned consultations and shifted nursing practice from reactive to anticipatory care [27]. However, these innovations require structured training to ensure oncology nurses can fully integrate them into practice.

### 4.2. Psychosocial and Emotional Support

Oncology nurses are increasingly recognized for their ability to deliver psychosocial care through virtual platforms. This includes providing emotional support, counseling, and education, as illustrated by the following examples. A systematic review conducted early in the COVID-19 pandemic found that telehealth counseling for cancer patients yielded non-inferior results compared to in-person sessions for psychosocial support [28]. Notably, virtual genetic counseling was found to be equally effective in improving patient knowledge and stress reduction. However, the review did not stratify its analysis by provider type, making it unclear whether oncology nurses specifically delivered these interventions.

The iCope intervention, a nurse-led psychoeducational telephone support program from McGill University, demonstrated significant reductions in uncertainty and anxiety (*p* = 0.006) among women undergoing breast cancer diagnostic at a Rapid Diagnostic Centre (RDC) [29]. The intervention consisted of two brief 15 min telephone sessions, reinforcing the feasibility of short, structured virtual support programs. However, the study faced recruitment challenges due to rapid RDC timelines, underscoring the need for better integration of virtual psychoeducational interventions within standard oncology nursing workflows.

Despite these promising findings, nurses report low confidence in addressing emotional needs virtually. An evaluation of virtual oncology care in Alberta found that while nurses were the most frequently engaged providers in virtual visits, only 31.5% felt confident in providing emotional support [5]. This again highlights the need for specialized training in digital communication to enhance oncology nurses’ capacity to deliver psychosocial care remotely.

Oncology nurses also frequently support family caregivers during virtual encounters, helping them navigate care plans, manage emotional distress, and reinforce patient education [30,31]. Virtual platforms can enhance family engagement, especially when caregivers are balancing multiple responsibilities or live at a distance [30,31]. However, virtual care also presents new challenges in assessing caregiver strain and providing relational support without the benefit of in-person cues [31].

### 4.3. Palliative and End-of-Life Care

Virtual palliative care models have emerged as essential components of oncology nursing, particularly for home-based and rural patients. A qualitative study in Ontario found that community palliative nurses coordinated virtual assessments, symptom management, and end-of-life care, often collaborating with physicians remotely to sustain hybrid care models [31]. These findings suggest that nurses play a central role in delivering virtual palliative services, ensuring continuity of care in remote and underserved settings.

Hybrid models that integrate in-person assessments with virtual support are increasingly recognized. Piazza and Drury (2023) highlighted how nursing-led telephone and virtual triage systems enable proactive symptom management, reducing unnecessary emergency visits [32]. However, their review also pointed to gaps in role clarity, digital literacy challenges, and a lack of standardized protocols, emphasizing the need for clear competencies in virtual palliative care.

### 4.4. Large-Scale Virtual Care Evaluations

The expansion of virtual oncology nursing has been evaluated at the system level, demonstrating feasibility and benefits in large-scale implementations. At Princess Margaret Cancer Centre in Ontario, over 22,000 virtual visits were conducted in two months, with nurses facilitating pre-visit triage, remote assessments, and patient follow-up. While 82% of patients and 72% of practitioners reported satisfaction with virtual care [15], the study did not evaluate nursing-specific experiences, training needs, or digital competency requirements—a gap that must be addressed in future research.

Similarly, a mixed-methods evaluation in Alberta highlighted the integral role of oncology nurses in virtual consultations, with nurses serving as the largest provider group. While 90% of patients expressed satisfaction with virtual oncology visits, particularly for follow-up care, the study identified critical gaps in emotional support, role definition, and digital training for nurses. Addressing these challenges is essential to optimizing nursing-led virtual oncology care, ensuring that nurses are equipped to provide comprehensive symptom management, psychosocial support, and patient-centered care in virtual settings [5].

A systematic review and meta-analysis further support the effectiveness of virtual nursing interventions for cancer patients [14]. The study analyzed eight randomized controlled trials (RCTs) comparing virtual nursing with traditional in-person care. Key findings indicate that virtual nursing interventions significantly improved patients’ quality of life (QoL), with a measurable positive impact (SMD = 0.22, 95% CI [0.01 to 0.43], *p* = 0.04). These interventions provided structured symptom monitoring and emotional support, effectively addressing pain, anxiety, and fear of recurrence. Additionally, virtual nursing enhanced accessibility to oncology care, particularly benefiting patients in rural or remote areas by reducing the need for frequent hospital visits while ensuring continuous support. However, the review also identified barriers to virtual nursing, including role ambiguity, digital literacy challenges, and the need for stronger nursing training programs to support virtual oncology care delivery.

### 4.5. International Perspectives

In addition to the aforementioned studies from Canada, France, and Belgium, international case studies further highlight the importance and adaptability of oncology nurses in virtual care. In Norway, oncology nurses used videoconferencing to deliver follow-up care and psychosocial support to rural patients, playing a key role in helping patients navigate the technology and manage symptoms remotely [33]. In the United States, nurses at Texas Oncology assumed new virtual triage responsibilities during the pandemic, providing proactive support and symptom management through telehealth platforms [34]. In the Global South, oncology nurses adapted to virtual care amid resource constraints, using telehealth to provide cancer support while navigating equipment shortages and workforce challenges [35]. This demonstrates both resilience and innovation in under-resourced settings. These global examples reinforce the widespread relevance of virtual oncology nursing and its potential to support equitable cancer care delivery worldwide.

This research demonstrates the importance and value of virtual care oncology nursing in patients’ psychosocial, emotional, and symptom care and management. This research also demonstrates the impacts of this approach on large patient groups (i.e., palliative care patients) and the impacts of virtual nursing care upon patient and organizational outcomes. Yet, there is much to be fully studied and understood regarding virtual oncology nursing roles, training approaches to facilitate competency development, and optimal approaches to virtual oncology nursing delivery models and their appropriateness depending on patient needs (i.e., when fully virtual or hybrid models should be used), within a broader context of patient and healthcare system virtual care processes and outcomes.

## 5. Call to Action

The evolving role of oncology nurses in virtual care must be reinforced through role definition, standardized competencies, professional training, and structural investments. Hybrid care models must be formally integrated into workforce planning, ensuring that nurses have the skills and resources to navigate both virtual and in-person oncology care effectively. As Dowling et al. (2023) emphasize, digitalization in cancer care is a top research priority, necessitating the alignment of nursing workforce development, education, and technology infrastructure to sustain high-quality virtual oncology care [36].

### 5.1. Defining the Role of Oncology Nurses in Virtual Care

Virtual oncology nursing has historically developed under physician-driven telemedicine models, limiting its recognition as an independent discipline. While nurses facilitate symptom management, patient education, and interdisciplinary coordination, they rarely lead independent virtual interventions [1]. The expansion of nurse-led virtual care models, such as remote patient monitoring (RPM) and digital symptom triage, requires clear role definitions to ensure oncology nurses are recognized as primary care providers in virtual oncology settings. Future models of virtual oncology care must consider how to better integrate family caregivers, ensuring that nurses have the tools and training to support not only patients but also their informal care networks through digital platforms.

### 5.2. Establishing a Standardized Educational Framework

Despite the growing integration of virtual care, nursing education has not kept pace with digital health advancements. Many telehealth roles recommend a minimum of three years of clinical experience [9], reflecting the absence of structured pre-licensure training in virtual nursing practice. Regulatory bodies such as CANO and CASN must collaborate to develop and integrate oncology virtual care competencies into certification programs and continuing education curricula.

Virtual oncology nursing education should include training in three key areas, though we acknowledge that the full integration of these competencies into nursing curricula is a complex challenge that warrants further research and dedicated development. First, telehealth communication strategies should be embedded in psychosocial and symptom management training. The iCope intervention offers one such example, where oncology nurses were trained to deliver brief, structured telephone-based psychosocial support that significantly reduced anxiety and uncertainty among patients undergoing diagnostic procedures [29]. Second, digital health literacy is essential for ensuring effective and confident use of virtual platforms. For example, Shi et al. (2024) showed that oncology nurses supporting patients in using a mobile symptom self-monitoring app helped reduce unplanned consultations and fostered anticipatory care [27]. Third, the Pan-Canadian Oncology Symptom Triage and Remote Support (COSTaRS) tools offer standardized telephone-based triage protocols that can be embedded into case-based learning and simulation within nursing education [25]. Embedding these competencies into both undergraduate and continuing education programs will require experiential learning opportunities, such as simulation, mentorship, and case-based activities, to support skill transfer into practice.

### 5.3. Building Supportive Models of Care

To ensure the sustainability of virtual oncology nursing, structured workforce planning and hybrid care frameworks are essential. To date, much of the work on nurse virtual care competencies has been limited, with the primary focus being on the development of entry-to-practice competencies for baccalaureate nurses. As outlined earlier in this commentary, there is a need to extend this work to develop specific competencies and training that are linked to current and future models of oncology nursing care, taking into account oncology nursing practice and oncology team and patient outcomes. This includes taking into account evidence [e.g., Knox et al. from British Columbia (2021) and Rodriguez et al. (2022)] that underscores the importance of data-driven staffing models that quantify nursing workload and align resources with virtual care demands [37,38]. The integration of AI-driven triage tools, RPM systems, and virtual multidisciplinary case reviews can streamline workflow and optimize patient outcomes.

### 5.4. Addressing Infrastructure and Technological Barriers

Persistent technological disparities pose challenges for both oncology nurses and patients. While virtual platforms have expanded access, lack of digital literacy, inconsistent electronic health record (EHR) integration, and security concerns remain barriers.

To strengthen virtual oncology care: (a) healthcare institutions must invest in secure, interoperable virtual care platforms; (b) training programs should focus on enhancing nurses’ comfort with digital tools; (c) policies must address inequities in technology access, particularly in remote communities [1]. In addition to this, there is a need to integrate and include nurses as stakeholders in technology procurements, user interface design, and workflow configuration involving virtual technologies and their implementation given the impacts of virtual care technologies upon nurse decision-making and patient outcomes. Researchers have identified the features and functions of telehealth systems that influence nurse decision-making and the quality of virtual care interactions [20,21]. They recommended the application of cognitive task analysis, cognitive screen turn analysis, usability testing, and clinical simulations for the optimization of software used in tele-nurse practice to improve nurse satisfaction with the technologies used, optimize the number of virtual care encounters, and prevent user errors associated with the technology. Nurse involvement in such technology optimization activities was identified as essential [20,21].

In addition to technical and operational limitations, virtual oncology care also raises important ethical considerations. Patients with cancer are often navigating complex and life-threatening conditions, and the shift to virtual platforms can introduce challenges related to equity, autonomy, and relational care. For example, limited digital literacy may compromise a patient’s ability to provide informed consent or engage meaningfully in care planning [7]. Geographic and socioeconomic disparities may further restrict access to high-quality virtual care in rural or under-resourced communities [4]. Moreover, standardized algorithms used in virtual symptom triage may insufficiently account for complex, evolving cancer trajectories and social vulnerabilities, therefore raising ethical concerns about bias, depersonalization, and inequity in virtual care [15]. These tools may inadvertently introduce bias by assuming linear or uniform symptom trajectories, which risks underrepresenting patients with atypical disease patterns, comorbidities, or social vulnerabilities. These limitations risk undermining the core ethical principles of personalized, compassionate care. Oncology nurses are uniquely positioned to identify and mitigate such ethical gaps by participating in the design and evaluation of virtual care platforms, advocating for equitable access, and preserving the humanistic elements of care through skilled digital communication [15,20,21].

## 6. Conclusions

Virtual oncology nursing has reached a pivotal juncture where the profession must transition from adapting to virtual care to actively shaping its future. Ensuring the long-term sustainability of digital health in oncology requires investment in nursing leadership, targeted digital health training, and structural integration of virtual care into standard practice.

Moving forward, the success of virtual oncology nursing will depend on redefining nursing leadership in virtual care, clarifying roles, fostering interprofessional collaboration, and developing hybrid models that blend in-person and virtual care seamlessly. Strengthening digital health competencies and expanding structured professional development opportunities will be key to empowering oncology nurses to lead in virtual cancer care.

## Abbreviations

The following abbreviations are used in this manuscript:

PRO	Patient-reported outcome
EHR	Electronic Health Record
CNA	Canadian Nurses Association
COSTaRS	The Pan-Canadian Oncology Symptom Triage and Remote Support
RPM	Remote patient monitoring
RCT	Randomized controlled trial
QoL	Quality of life
